# Expert Views on Criteria for Evaluation of Human Factors Methods: Qualitative Interview Study

**DOI:** 10.2196/73324

**Published:** 2026-02-03

**Authors:** Selvana Awad, Rachel Begg, Thomas Loveday, Andrew Baillie, Melissa Baysari

**Affiliations:** 1 The University of Sydney, Camperdown, Sydney, Australia; 2 NSW Health, 1 Reserve Rd, St Leonards, 2065, Australia; 3 Sydney Local Health District, Camperdown, Australia

**Keywords:** human factors, evaluation, methods, quantitative, qualitative, framework

## Abstract

**Background:**

Human factors (HF), or ergonomics, which explores the interaction between humans and systems, has been used to support design in safety-critical industries such as aviation, transportation, nuclear power, and manufacturing. HF methods have the potential to support the safe design of health IT; however, the evaluation of HF methods to determine their effectiveness and feasibility in this context has been limited.

**Objective:**

The aim of this study was to identify criteria for evaluating HF methods when applied to real-world projects and to use these to propose a framework for method evaluation.

**Methods:**

The study design was qualitative and descriptive and involved semistructured interviews with HF experts working across health and nonhealth industries in academic and/or practitioner roles. HF experts held a relevant degree (eg, ergonomics and HF engineering) and were actively using their HF expertise. Results were thematically analyzed.

**Results:**

A total of 21 participants took part, and interviews lasted, on average, 52 (range 39‐103) minutes. Participants mentioned that they did not routinely evaluate methods; however, when asked how they would evaluate methods, they outlined a range of criteria to support method evaluation. Overall, 5 criteria and 28 subcriteria were identified. High-level criteria included effectiveness, efficiency, ease of use and acceptability, and impact on the solution.

**Conclusions:**

Results from this study were used to propose a framework for evaluating HF methods used in real-world health IT projects. The framework should provide organizations with valuable information on how to optimize the application and outcomes of HF methods and build HF capability within organizations, particularly where this capability may be lacking.

## Introduction

Health IT (HIT) enables the processing, storage, and exchange of health information in an electronic environment [1]. In the United States, the Meaningful Use program has led to a significant increase in the adoption of HIT, such as electronic health records [1]. Although clear benefits of HIT have been demonstrated [2,3], research has shown that many HITs suffer from poor usability [4,5]. Usability is a measure of how well a specific user in a specific context can use a product to achieve a defined goal safely, effectively, efficiently, and satisfactorily [5]. Usability can be compromised by poor design and lead to “use errors” [5-7]. The relationship between system design and safety in the context of HIT is well recognized in the literature [5,8-12]. For example, a recent systematic review found that poorly designed electronic health records were associated with usability issues such as poor data entry, lack of workflow support, and inadequate automation [13]. These issues directly contributed to medication errors, such as patient overdoses, and had other negative impacts on medication safety [13]. Human factors (HF) methods have the potential to reduce “use errors” and, in turn, improve patient outcomes [7].

The discipline of HF, or ergonomics, which explores the interaction between humans and systems, has been used to support design in safety-critical industries such as aviation, transportation, nuclear power, and manufacturing [7,14-16]. While some HF methods, particularly those focused on human-computer interaction (eg, usability testing), have been used to support the design and redesign of HIT, the use of safety-focused and systems-based HF methods in this context is limited [8,17]. HF methods commonly used in the HIT context tend to have a linear and micro perspective of potential issues that may affect system use and user behavior, as they focus on specific problems that may be encountered by the individual user using the system rather than considering the entire sociotechnical system [8,17,18]. HF methods that apply a systems thinking lens to identify problems and risks that may arise from the interactions between components of a complex system are less commonly used in HIT design and evaluation [8,17,19,20].

Potential reasons for the limited use of HF methods include issues with the availability of HF expertise in health care, a research-practice gap, a potential echo chamber effect within health care whereby the same subset of well-known methods is used repeatedly, issues with method usability, and challenges with demonstrating the value added by HF application to justify up-front investment [10,17,20,21]. Further research is required to demonstrate the value of applying HF methods (particularly those with a systems safety focus) to HIT, supported by a robust evaluation approach or framework [22,23].

Evaluation of HF methods to determine their effectiveness and feasibility has been limited. Previous evaluations have mainly been in the context of academic studies evaluating the reliability, validity, and efficacy of some HF methods, for example, when applied by health care participants as compared with experienced HF experts [20]. Despite these studies, empirical data regarding the reliability or validity of many HF methods do not exist [24]. Several challenges associated with conducting these forms of evaluations have been cited, including challenges with recruiting enough experts to enable comparison with a gold standard, time and resource intensiveness, and limited knowledge of appropriate statistical analyses [22,24].

An additional challenge is that there is no consensus or agreement on what constitutes an effective or valuable HF method. How do HF practitioners select and evaluate methods when applying them to real-world projects as part of HF integration processes?

Although a previous paper provides suggestions on potential evaluation criteria for HF methods [18], these are not comprehensive and represent the authors’ recommendations rather than findings derived from research methods. The aim of this study was to identify criteria for evaluating HF methods when applied to real-world projects and to use these to propose a framework for method evaluation.

## Methods

### Overview

This qualitative descriptive study was undertaken as part of a larger study that focused on HF and safety analysis methods for use in the design, redesign, and configuration of HIT. The study design involved semistructured interviews with HF experts working across health and nonhealth industries in academic and/or practitioner roles. A participant was considered an HF expert if they had a relevant degree (eg, ergonomics and HF engineering) and were actively using their HF expertise. Part 1, currently under review, explored what HF methods experts use and how they are selected. Part 2, reported here, explored criteria HF experts use or view as important to evaluate HF methods. Questions used to guide the semistructured interviews were developed by a clinical informatics professional with expertise in design, HF, and safety and quality (SA) and a HF expert (MB) and reviewed by other members of the research team with HF (TL and RB) and implementation science (AB) expertise. After collecting demographic information, the 2 main questions asked relevant to this study were “What makes a ‘good’ HF method to you?” and *“*How would you evaluate a HF method?*”*

Participants were recruited through a combination of purposive, opportunistic, and snowball sampling. Recruitment involved advertising the study through national HF societies and relevant working groups; promotion at an international HF and patient safety conference through networking and word-of-mouth approaches; consultation with HF experts and contacts to recommend potential participants; and a review of common HF textbooks and literature to identify HF authors who could be invited to participate. In addition, HF practitioners known to the research team were directly approached. Suitable participants interested in the study, including those identified by the investigators, were invited to take part in the study via email. Recruitment continued until thematic saturation was reached, that is, no new themes were emerging from the data [25].

The interviews, conducted by SA, occurred via videoconferencing, except for one, which was face to face. Deidentified content from the interview transcripts was independently analyzed by 2 investigators with expertise in HF and qualitative analysis (SA and RB) using a general inductive approach [26]. Each investigator independently coded data for the first 5 interviews and then met to discuss findings and reach consensus on a high-level framework to support the documentation of codes into themes and subthemes. For the remainder of the interviews, each investigator continued to independently assign text to the agreed themes and subthemes using the framework and, at the end of this process, met to discuss any further discrepancies until consensus was reached [27]. Overall, the 2 investigators were generally consistent in their coding and identification of themes and subthemes. Disagreements were minor and resolved via discussion until consensus was reached.

### Ethical Considerations

Ethics approval was obtained from the University of Sydney’s Human Research Ethics Committee. All participants provided informed consent and agreed to be audio-recorded. All transcripts were de-identified prior to data analysis. All participants provided informed consent and agreed to be audio-recorded.

## Results

### Participant Demographics

A total of 21 participants took part, and interviews lasted, on average, 52 (range 39‐103) minutes. Table 1 describes the demographics of the 21 participants.

**Table 1.** Characteristics of the participants included in the interviews (N=21).

Core industry and core role		Participants, n	Within industry category, %	Within total participants, %
Health (n=14)				
	Academic	9	64	43
	Practitioner	2	14	10
	Academic and practitioner	3	21	14
	Total	14	100	67
Nonhealth (n=7)				
	Academic	2	29	10
	Practitioner	5	71	24
	Academic and practitioner	0	0	0
	Total	7	100	33

### How Should HF Methods Be Evaluated?

Participants explained that they did not routinely evaluate methods; however, when asked how they would evaluate them, they outlined a range of criteria to support method evaluation (Table 2). High-level criteria included effectiveness, efficiency, ease of use and acceptability, and impact on the solution. Other criteria, such as validity, reproducibility, and reliability, were mainly mentioned by academic participants rather than practitioners. Overall, 5 criteria and 28 subcriteria were identified.

**Table 2.** Criteria for human factors (HF) method evaluation, as reported by participants.

Criteria and subcriteria	Example quotes
Effectiveness	
1.1 Effectiveness in identifying usability issues	[N]ice to see whether or not the amount of usability issues related to patient safety. [P2]
1.2 Effectiveness in identifying safety issues	[N]ice to see whether or not the amount of usability issues related to patient safety. [P2] ...there’s a primary metric that matters most like which method uncovered the most safety issues in advance of implementation or which method uncovered the more severe safety issues [P9]
1.3 Ability to achieve intended impact, change or goal	What value did it add? Like? Do you feel like added value to the project? Do you feel like it gave you the right answers or the right tools to get to the answers that you wanted? [P16] Does it make the change that you envision?...And that has to do with your final goal [P6]
1.4 Generates recommendations that are useful and easy to implement	Does it generate recommendations [that] are easy to implement? And that then do [they] get implemented and lead to risk management? [P7] [T]he other thing that’s really important to us in terms of criteria is going beyond analysis and figuring out a method that can actually produce useful information, and actionable information for redesign for change, for implementation, whatever you want [P11]
1.5 Ability to identify micro, meso and macro (systems) level considerations that have safety implications	[Y]ou might do an overarching review of the technology in the context of the people using it in the context of the work in the workplace and then on the basis of that, deploy specific things that are the micro, meso, or macro level, to understand a bit more [P7]
1.6 Method’s effectiveness in understanding the dynamic nature of the system	And another important thing is to continue to collect data about the use of the system, what I call the dynamic safety of an application. [P13]
1.7 Ability to cover the required domain areas/constructs (eg, usability, safety, workload, situational awareness, and decision-making)	And then I think it’s the core human factors constructs. It’s kind of like, what’s the impact on situational awareness, workload? Usability, and decision making, are probably some key constructs that you might want to check [P21]
1.8 Overall usefulness	[T]he other thing that’s really important to us in terms of criteria is going beyond analysis and figuring out a method that can actually produce useful information, and actionable information for redesign for change, for implementation, whatever you want [P11]
Efficiency	
2.1 Demand on time and resources (e.g.eg, workload)	How fast it was... how much time [and] how much budget it came under. [P16]
2.2 Cost required	Is it cost effective? [P6]
2.3 Overall efficiency	It’s to look at the efficiency of the methods [P4]
Ease of use and acceptability	
3.1 Ease of use	Is it usable, based on some objective standard usability? Or is it more usable than the alternative? [P7] [Y]our method should be easy to use... not too demanding in human resources in training, and not too costly [P1] [E]ase of use of the method and the ease of understanding the tools from the people who participate [P13]
3.2 Learnability	How quick it was to learn [P16]
3.3 Utility and acceptability of the method	The types of questions that make sense are acceptability. You know, utility...in [a] very basic sense, we applied this in this way, what did we learn, we learned something new [P7]
3.4 Overall satisfaction of those applying the method	[E]ase of use of the method and the ease of understanding the tools from the people who participate [P13]
3.5 Participant experience	[E]ase of use of the method and the ease of understanding the tools from the people who participate [P13] [W]e asked participants to give us feedback on the method...I think that’s an important source of feedback and evaluation of the method, what do they think about it? [P11]
3.6 Likely adoption of the method based on complexity and learnability	If you develop a very complex method, it may be the best one to identify all the problems that could lead to a risk for the patient. But...nobody’s applying it [P1]
3.7 Adoption of the method by non-HF experts	[T]alked about giving away ergonomics. And in a lot of the things that we’ve done that has been our goal when we leave, can they actually do things on their own? Even if it’s a little bit more simple than the way we would do it? [P11]
3.8 Whether the method met expectations	And another important thing probably is to clarify, the time needed to apply one method and the expected outcome, like a sort of table of the expectation that one can have through the application of that method, because too often, people look at the ergonomist, either those with the silver bullet in the hand, or those with some annoying requirements to be applied in the design process. [P13]
3.9 Ability of the method to be adapted	[D]evelop questions that can be added into evaluation about the potential adaptation of these methods [P7]
Impact	
4.1 Impact on patient safety and other outcomes	[If] you’ve got a goal of patient safety...the main criterion should be the risk to the patient... the likelihood of patient safety issues...if you want that your method is used, if you want to make it to be used by vendors and hospital later...analyze their human factors or risk, relate the human factors related risks of EHR [electronic health record] [P1] Is the whole system in total more safe? [P6]
4.2 Impact on end user workflows and workloads	[Y]ou can also identify workload issues that will slow down the care process even if it doesn’t threaten directly patient [P1]
4.3 End user satisfaction (of the health information technology)	The satisfaction of the end users also could be analyzed [P1]
4.4 Adoption of the system or tool by end users	Is it used? Is it actually used? By all your users? [P6]
4.5 Impact on redesign (ie, whether changes or improvements were made)	So when we redesigned the document, how many of those many, many vulnerabilities or problems were identified in the document? How many of those were we able to address? So, you know, having concrete impact on design, I think is a big issue. [P11]
Other	
5.1 Preestablished validity	I think these reliability and validity studies are really important [P7]
5.2 Reproducibility of results	[D]id you align to see if you meet that standard? ...and then do all these things that are for measurements, I think it must be reproducible [P9]
5.3 Reliability if applied by different people (interrater reliability)	I think these reliability and validity studies are really important [P7]

Participants commented on challenges with quantitatively evaluating methods using the criteria identified and suggested a more qualitative, self-assessment–based evaluation; for example:

*We usually sit down when we’ve concluded a project as part of the HF team and we will talk about the methods we used and how we feel they performed, whether we had any pitfalls, whether we wished we had something different, or we’ve done something different.*

[P16]

## Discussion

This study identified 5 high-level criteria and 28 subcriteria to support the evaluation of HF methods in real-world HIT projects, as reported by HF practitioners across health and nonhealth industries.

Although previous studies have evaluated HF methods by focusing on validity and reliability [23], this study is the first of its kind to identify a broad range of evaluation criteria for HF methods, as recommended by HF practitioners. There is some overlap between our criteria and those developed by Waterson et al [18], which focused on the evaluation of systems-based methods. As such, this study adds to findings generated by Waterson et al [18] by confirming some of the criteria identified in this previous work. This includes outcomes of the method, the method’s robustness, the method’s usability and support requirements, aspects related to work domains, and aspects related to different levels (ie, individual, team, and organizational) [18].

Our study adds to this by identifying new criteria, particularly those related to operationalized usability, adoption, and other impacts on users (eg, workload); outcome-focused effectiveness, including the link between usability and safety; and more nuanced consideration of system-level considerations (micro, meso, and macro). Furthermore, the findings from our study offer a combined quantitative and qualitative approach to evaluation, with a focus on actionable insights and bridging theory and practice. These points highlight the value of exploring the views of a range of HF experts working across different industries [18].

Our study also elaborates on the method’s effectiveness in understanding the dynamic nature of the system, which is aligned with systems safety thinking that defines safety as a dynamic, emergent property of how system components interact with each other [19,28-30]. A dynamic system is a complex and adaptive system that changes behavior due to interactions between system components [31-33]. As such, the safety of a system can change over time and is impacted by many variables, such as human performance, resources, and events at particular points in time. This is consistent with a study that aimed to evaluate methods, such as the functional resonance analysis method, using resilience characteristics as indicators of core safety factors [34].

Participants commented on challenges with quantitatively evaluating methods and suggested that a more qualitative approach could be used. This is generally aligned with other literature that suggests that outcome-based quantitative evaluation is not widespread and can be resource intensive [18]. Furthermore, demonstrating the impact of methods can be difficult, as system usability and safety are influenced by a range of factors outside of the method itself [18]. While many factors influence outcomes, health organizations may still benefit from using measures, for example, the number of usability and safety issues identified and user satisfaction scores, to evaluate whether applying methods results in detection of usability and safety issues. Where possible, comparative evaluation to demonstrate that applying methods is better than not applying any method may also be of value. Although such approaches may lack the academic rigor of reliability and validity studies, they may increase an organization’s degree of confidence in HF methods and, therefore, willingness to invest in them. Such evaluation may help health staff and managers develop business cases for the application of HF, help organizations reflect on strategies to enhance the use of methods (eg, organizational support), and refine when and how HF methods are applied to deliver the most value within the context of challenging and complex HIT projects. This type of evaluation could occur through self-reported Likert-scale ratings (as done by Waterson et al [18]); reflective qualitative discussions, as suggested by participants in this study; and other relevant metrics.

This study had several limitations. The recruitment process relied on interested experts volunteering to participate, so the sample may have been biased and our results may not represent the views of all HF experts. We included both health and nonhealth participants, as well as practitioners and academics, but did not compare the views of participants from different groups. Although our sample size was modest, we continued interviews until thematic saturation was reached, which is the norm in qualitative research.

**Figure 1.**
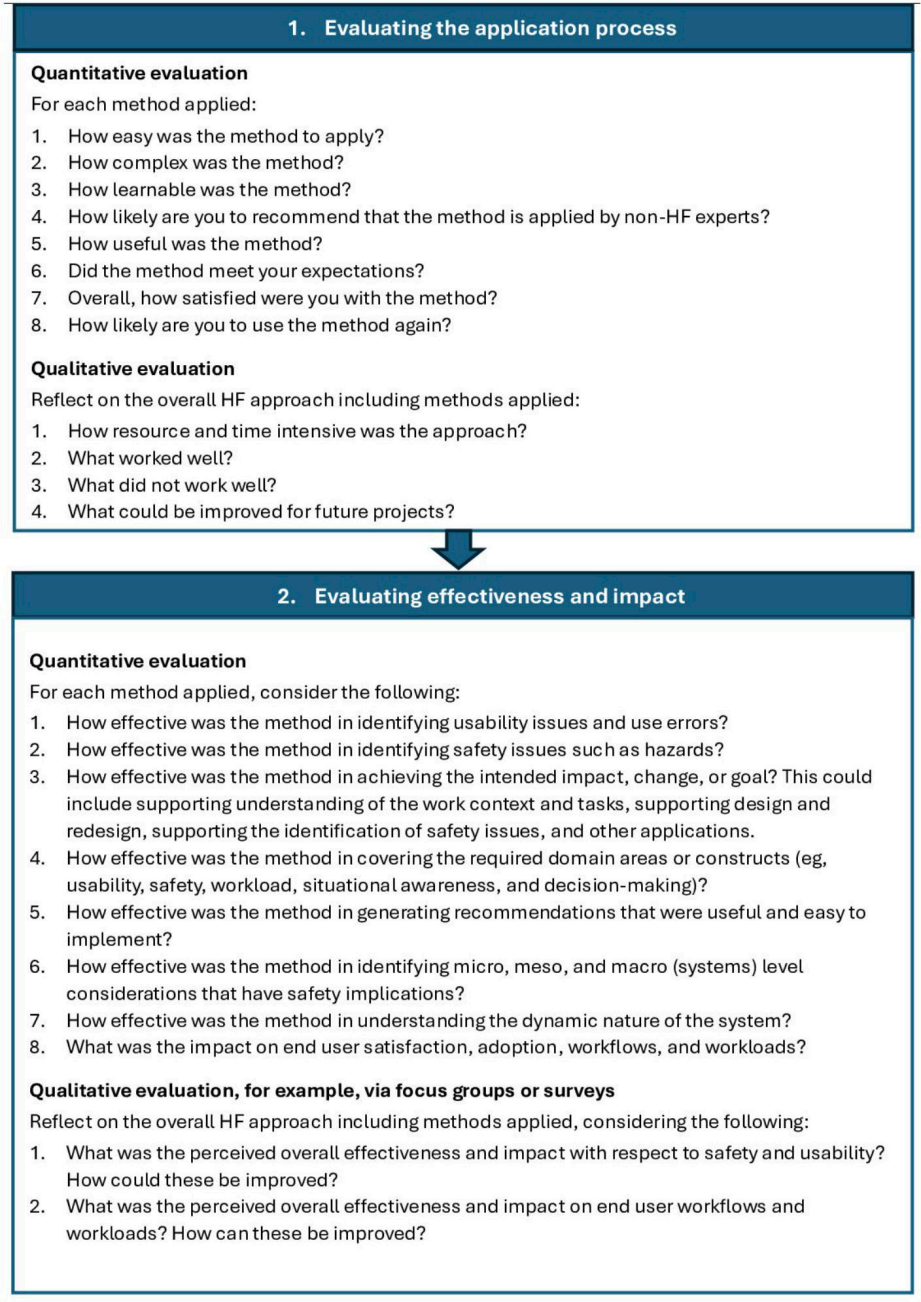
Proposed framework for evaluating human factors (HF) methods.

We used participant responses and systems safety knowledge to propose a framework for evaluating HF methods in the context of real-world HIT projects (Figure 1). By covering both process- and outcome-based evaluation, this framework loosely aligns with other evaluation frameworks, such as those used in quality improvement and program evaluation [35,36]. While we also recommend further reliability and validity academic studies to validate the robustness of HF methods in the HIT context, our proposed framework offers a subjective yet structured approach for HF method validation that can be applied by organizations to real-world HIT projects where HF methods have been used. The framework, which we recommend be applied flexibly depending on the nature of the project and the resources available, should provide organizations with valuable information on how to optimize the application and outcomes of HF methods and build HF capability within organizations, particularly where this capability may be lacking.

## Abbreviations

HF: human factors
HIT: health IT
